# Thyroxine changes in COVID-19 pandemic: A systematic review and meta-analysis

**DOI:** 10.3389/fendo.2023.1089190

**Published:** 2023-02-13

**Authors:** Ziqi Li, Pengwei Hou, Shuwen Mu, Renzhi Wang, Hui Miao, Ming Feng, He Wang, Wentai Zhang, Yihao Chen, Tianshun Feng, Shousen Wang, Yi Fang

**Affiliations:** ^1^ Department of Neurosurgery, Dongfang Affiliated Hospital of Xiamen University, School of Medicine, Xiamen University, Xiamen, China; ^2^ Department of Neurosurgery, Fuzong Clinical Medical College of Fujian Medical University, Fuzhou, China; ^3^ Department of Neurosurgery, 900TH Hospital of Joint Logistics Support Force, Fuzong Clinical Medical College of Fujian Medical University, Fuzhou, China; ^4^ Department of Neurosurgery, Peking Union Medical College Hospital, Chinese Academy of Medical Sciences, Peking Union Medical College, Beijing, China

**Keywords:** free triiodothyronine (FT3), free thyroxine (FT4), thyroid stimulating hormone (TSH), COVID-19, severity (S)

## Abstract

**Objective:**

COVID-19 infection may affect thyroid function. However, changes in thyroid function in COVID-19 patients have not been well described. This systematic review and meta-analysis assess thyroxine levels in COVID-19 patients, compared with non-COVID-19 pneumonia and healthy cohorts during the COVID-19 epidemic.

**Methods:**

A search was performed in English and Chinese databases from inception to August 1, 2022. The primary analysis assessed thyroid function in COVID-19 patients, comparing non-COVID-19 pneumonia and healthy cohorts. Secondary outcomes included different severity and prognoses of COVID-19 patients.

**Results:**

A total of 5873 patients were enrolled in the study. The pooled estimates of TSH and FT3 were significantly lower in patients with COVID-19 and non-COVID-19 pneumonia than in the healthy cohort (P < 0.001), whereas FT4 were significantly higher (P < 0.001). Patients with the non-severe COVID-19 showed significant higher in TSH levels than the severe (I^2^ = 89.9%, *P* = 0.002) and FT3 (I^2^ = 91.9%, *P* < 0.001). Standard mean differences (SMD) of TSH, FT3, and FT4 levels of survivors and non-survivors were 0.29 (*P*= 0.006), 1.11 (*P* < 0.001), and 0.22 (*P* < 0.001). For ICU patients, the survivors had significantly higher FT4 (SMD=0.47, *P*=0.003) and FT3 (SMD=0.51, P=0.001) than non-survivors.

**Conclusions:**

Compared with the healthy cohort, COVID-19 patients showed decreased TSH and FT3 and increased FT4, similar to non-COVID-19 pneumonia. Thyroid function changes were related to the severity of COVID-19. Thyroxine levels have clinical significance for prognosis evaluation, especially FT3.

## Introduction

COVID-19 pneumonia is caused by SARS-CoV-2 and broke out in 2019, causing unprecedented issues worldwide. SARS-CoV-2 infection can trigger systemic inflammatory symptoms involving systemic multi-organ multisystem dysfunction (1, 2). Since the pandemic outbreak, several studies have demonstrated varying degrees of impaired thyroid function in patients with COVID-19 (3, 4). Viral infections may affect thyroid function through hormones and immunoregulatory signaling molecules. However, changes in thyroid function in patients with COVID-19 have not been well described, and the particular mechanism is still controversial. Some mechanisms proposed are the virus’s direct or indirect invasion of the thyroid gland, effects of systemic inflammatory immune responses, and nonspecific adaptive mechanisms (5, 6). Previous studies have examined whether thyroid diseases increase the risk of adverse outcomes in patients with COVID-19. Some studies concluded that thyroid diseases do not affect the progression of COVID-19, whereas some reported poor outcomes in patients with COVID-19 and thyroid diseases (7, 8). Several narrative and systematic reviews have revealed conflicting results about the relationship between thyroid and COVID-19, arguing that thyroid diseases are unrelated to SARS-CoV-2 infection and deterioration (9–11). Conclusions may be controversial due to the lack of large-scale clinical studies. This study aims to evaluate available evidences systematically, assess the level of thyroid function in patients with COVID-19 through meta-analysis, and analytically compare differences in thyroid function among different populations during the epidemic. Non-COVID-19 pneumonia patients and healthy people during the epidemic were included in the study as controls.

## Methods

### Protocols and registration

Our methods were based on the Preferred Reporting Items for Systematic Reviews and Meta-Analyses (MOOSE) guidelines (**eMethods 1 in the Supplement**)and were registered in the International Prospective Register of Systematic Reviews (PROSPERO) database. The protocol number is CRD42022346568.

### Search strategy

We systematically searched PubMed/MEDLINE, Cochrane Reviews, Cochrane Central Register of Controlled Trials (Central), Web of Science and Embase databases in August 2022 without language restrictions. All published articles related to thyroid-related hormones and COVID-19 were searched. The literature search strategy was based on the following keywords: ([T3 OR FT3 OR triiodothyronine] or [T4 OR FT4 OR thyroxine] or [TSH or thyrotropin]) and (COVID-19 OR SARS-CoV-2 OR 2019 novel coronavirus). We then performed a manual search of studies meeting our inclusion criteria to identify articles apart from those found in the electronic databases. Two independent reviewers (ZL and PH) performed the first step of title/abstract screening and the second step of full-text assessment in the search process, and any disagreement that arose during this process was discussed until an agreement was reached.

### Study selection

We included observational studies in China and English language, to evaluate the correlation between COVID-19 disease and thyroxine levels. The complete list of articles obtained through the systematic search was screened to remove duplicates and exclude ineligible articles, including reviews, case reports, and studies with less than 20 patients. According to the inclusion or exclusion criteria, the full texts of all potentially qualified studies were independently reviewed by two reviewers (ZL) and (PH). Disagreements were addressed through discussion. A third reviewer (SM) resolved disagreements when a consensus could not be reached.

### Main outcomes and measures

The primary analysis assessed thyroid function in COVID-19 patients, comparing non-COVID-19 pneumonia and healthy cohorts. Secondary outcomes included different severity and prognoses of COVID-19 patients. Thyroxine levels of follow-up were also included.

### Data extraction

Basic Information, including author, country, type of study, sample size, mean or median age, sex ratio, and primary outcomes, such as death, severity, and survival, was extracted from the selected studies. The levels of thyroid hormones (FT3, FT4, and TSH) were extracted from patients in acute admission, survivors during follow-up, and deceased patients, in addition to those of healthy people and non-COVID-19 pneumonia patients during the pandemic. All extracted data were tabulated, and indexes measured by each research center were converted and unified.

We extracted data using standardized data abstraction forms. In case of missing data needed to conduct our meta-analyses, we contacted the authors, with a reminder 2 weeks later. Non-published data obtained from authors by communication are mentioned in the results section below, as applicable, with permission, and authors who responded are listed in Acknowledgments section.

### Quality assessment

Two reviewers independently assessed the risk of biases, including selection, performance, detection, attrition, and reporting biases, rated as low, high, or unclear risk. The quality of the included studies was evaluated using the Newcastle–Ottawa scale (**eTables 1, 2 in the Supplement**). The scale has a score of nine, and a seven or higher indicates high quality. Two reviewers performed data extraction and quality assessment independently, and a third reviewer checked the results. Disagreements were resolved by discussion.

### Statistical analysis

Continuous data (thyroxine levels: TSH, FT3, and FT4) were synthesized using mean difference (MD) with standard deviation from each study for the calculation of the average MD with a 95% confidence interval (CI). We applied Wan’s formula to estimate the relative means and standard deviations when continuous data were presented as medians and ranges (12). Adjusted SMD based on corrected data and other potential confounders were also presented. Meta-analyses were performed using the inverse variance method with the random-effects model when heterogeneity was statistically significant. The DerSimonian-Laird method with the fixed-effect model was used when heterogeneity was not statistically significant. Heterogeneity was assessed using Cochrane Q-test and I^2^ statistic, and a p-value of <0.05 indicated statistically significant heterogeneity. According to the Cochrane Handbook for Systematic Reviews of Interventions, the ranges of interpretation for I^2^ are as follows: 0%–40%, unimportant; 30%–60%, moderate heterogeneity; 50%–90%, substantial heterogeneity; and 75%–100%, considerable heterogeneity. All analyses were conducted using RevMan (version 5.4.1).

## Results

**Figure 1:**
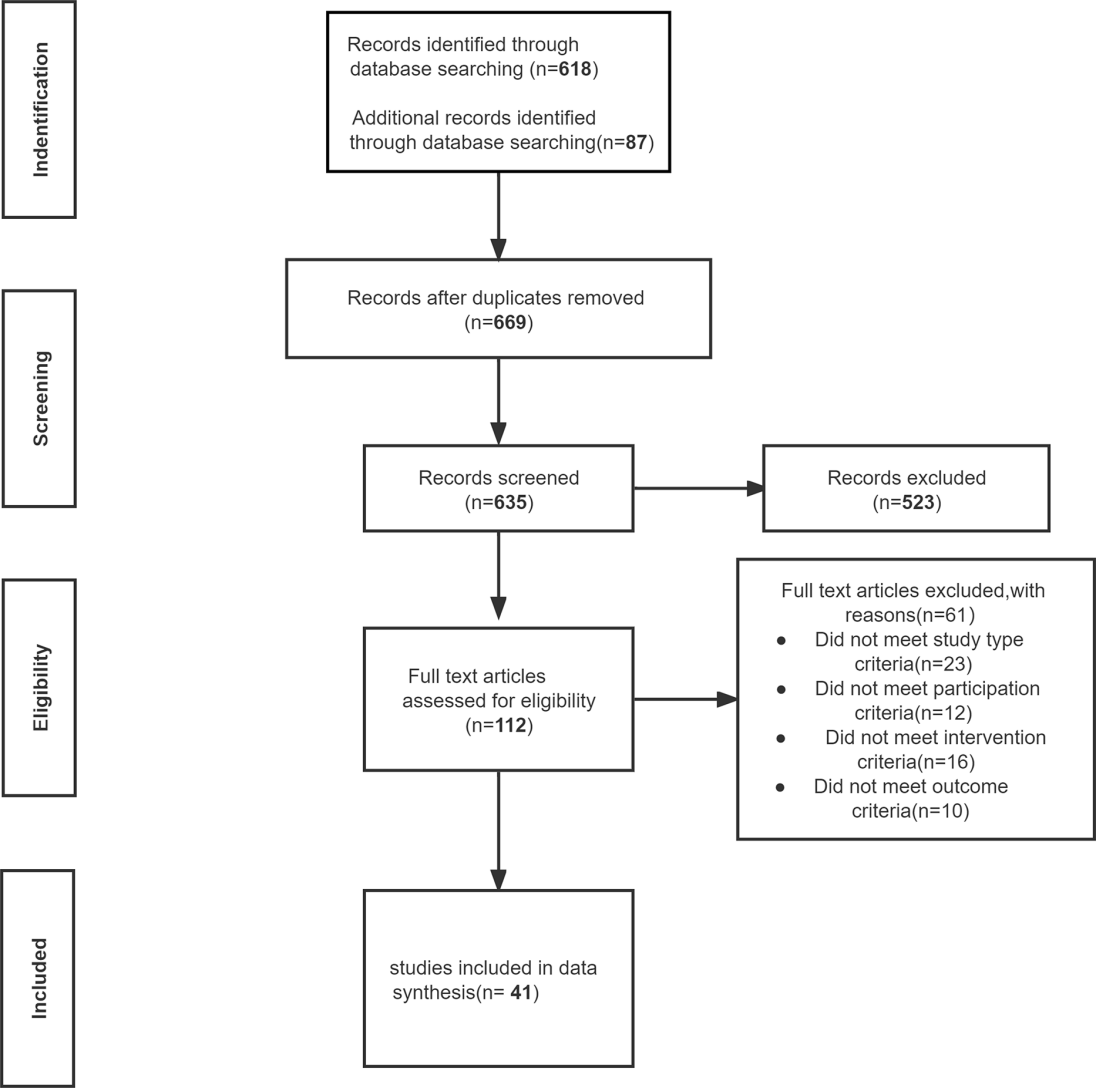
Flow diagram of the study selection process.

**Figure 2:**
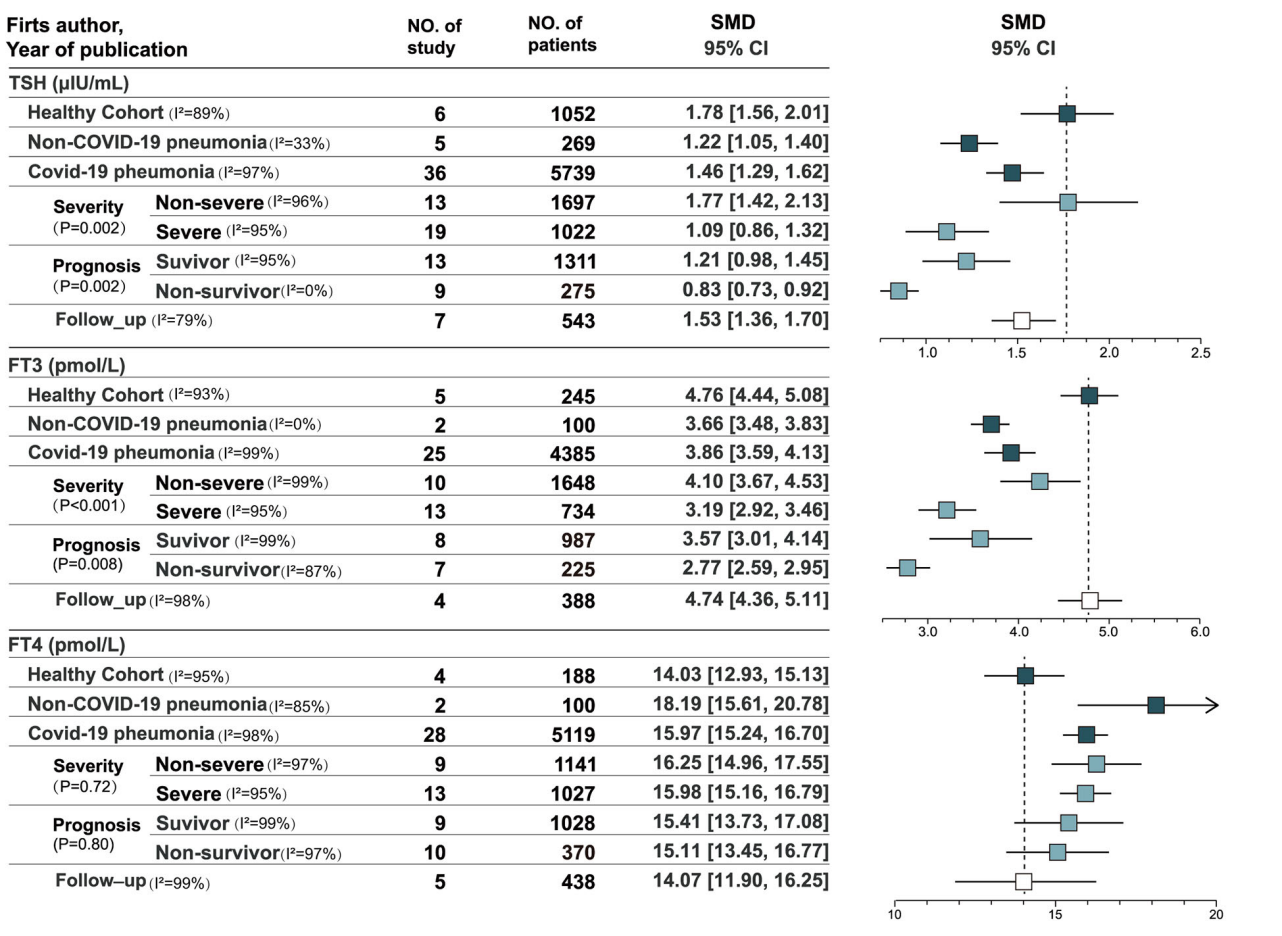
Forest plot for all studies.

The search time was up to August 2022, 635 relevant articles were screened, and 523 articles were excluded according to the titles and abstracts (**Figure 1**). The full texts of 112 articles were reviewed. Finally, 41 articles from 38 studies were included in the final analysis, totaling 5873 COVID-19 patients (13–38) (**Figure 2**). The control group included 269 patients with non-COVID-19 pneumonia and 1052 healthy people during the epidemic. The Characteristics of the included studies are presented in **Table 1**.

Table 1Description of eligible studies reporting the association between thyroid-related hormones and COVID-19.

**Table:** 

Author	Country	No. patients	Sex	Average age	Severity	Outcome	Control group	Thyroxine
Ahn (2021)	Korea	119	Male: 62	64	Non-severe: 32	Survivor: 85		TSH FT4
			Female: 57		Severe: 87	Non-survivor: 34		FT4
Ardes (2021)	Italy	118	Male: 64	73	Non-severe: 75	Survivor: 92		TSH
			Female: 54		Severe: 43	Non-survivor: 26		
Assimakopouls(2021)	Greece	22	Male: 11	62	Non-severe: 13		Healthy cohort: 19	TSH
			Female: 11		Severe: 9		Non-COVID-19 pneumon ia: 19	FT3
								FT4
Baldelli (2021)	Italy	46	Male: 32	60	Non-ICU: 23			TSH
			Female: 14		ICU: 23			FT3
Beltrao (2021)	Brazil	245	Male: 145		Non-critical: 181	Survivor: 204		TSH
			Female: 100		Critical: 64	Non-survivor: 41		FT3
								FT4
Campi (2021)	Italy	73			ICU: 73	Survivor(ICU): 57		TSH
						Non-survivor(ICU): 16		
Chen (2020)	China	274	Male: 171	59		Recover: 161		TSH
			Female: 103			Death: 113		FT3
Chen (2021)	China	50	Male: 33	48	Non-severe: 15	Survivor: 50	Healthy cohort: 54	TSH
			Female: 21		Severe: 35		Non-COVID-19 pneumonia: 50	
Clarke (2021)	UK	70	Male: 47	56	Non-severe: 42	Survivor: 70		TSH
			Female: 23		Severe: 28			FT3
								FT4
Clausen (2021)	Denmark	116	Male: 44	71		Survivor: 82		TSH
			Female: 72			Non-survivor: 34		FT4
Dabas (2021)	India	164	Male: 107	41	Non-severe: 100			TSH
			Female: 57		Severe: 64			FT3
								FT4
Das (2021)	India	84	Male: 42	41	Non-severe : 499			TSH
			Female: 42		Severe: 35			FT3
								FT4
Dutta (2021)	India	236	Male: 159	54	Non-severe: 200	Survivor: 225		TSH
			Female: 77		Severe: 36	Non-survivor: 11		FT3
								FT4
Gao (2021)	China	100	Male: 52	63	Non-sever: 34	Survivor: 44		TSH
			Female: 48		Severe: 66	Non-survivor: 22		FT3
								FT4
Gong (2021)	China	150	Male: 81	70	Non-sever: 25	Survivor: 118		TSH
			Female: 69		Severe: 125	Non-survivor: 42		FT3
								FT4
Grondman (2021)	Netherlands	161	Male: 56	65	Non-ICU: 120	Survivor: 141		TSH
			Female: 105		ICU: 41	Non-survivor: 20		FT4
Gliven (2021)	Turkey	250	Male: 157	67	Non-ICU: 125	Survivor(ICU): 88		TSH
			Female: 93		ICU: 125	Non-survivor(ICU): 37		FT3
								FT4
Khoo (2020)	UK	334	Male: 203	66		Survivor: 239		TSH
			Female: 131			Non-survivor: 95		FT4
Kumar (2021)	India	235	Male: 147	49	Non-severe: 202	Survivor: 222		TSH
			Female: 88		Severe: 33	Non-survivor: 13		FT3
								FT4
Lang (2021)	China	127	Male: 62	64	Non-severe: 56	Survivor: 116		TSH
			Female: 65		Severe: 71	Non-survivor: 11		FT3
								FT4
Li (2020)	China	40		44	Non-severe: 40		Healthy cohort: 57	TSH
								FT3
								FT4
Lui (2020-2021)	China	541	Male: 245	50	Non-severe: 499	Survivor: 283		TSH
			Female: 196		Severe: 42			FT3
								FT4
Malik (2021)	Pakistan	48	Male: 31	51	Non-severe: 22	Survivor: 48	Non-COVID-19 pneumonia: 28	TSH
			Female: 17		Severe: 26			
Nakamura (2021)	Japan	147	Male: 95	70	Non-severe: 63			TSH
			Female: 52		Severe: 84			FT3
								FT4
Okoye (2022)	Italy	95	Male: 50	82		Survivor: 70	Non-COVID-19 pneumonia: 81	TSH
			Female: 45			Non-survivor: 25		FT3
								FT4
Okwor (2021)	Nigeria	45	Male: 95	35			Healthy cohort: 45	TSH
			Female: 52					FT3
								FT4
Schwarz (2021)	Israel	54	Male: 37	59	Non-ICU: 37	Survivor: 44		TSH
			Female: 17		ICU: 17	Non-survivor: 10		FT3
								FT4
Sciacchitano (2021)	Italy	62	Male: 29	67				TSH
			Female: 33					FT3
								FT4
Sen (2021)	India	60			Non-severe: 42			TSH
					Severe: 18			FT3
								FT4
Sparano (2022)	Italy	506	Male: 315	69	Non-severe: 506			TSH
			Female: 191					FT4
Urhan(2022)	Turkey	64	Male: 32	39	Non-severe: 38	Survivor: 64	Healthy cohort: 70	FT3
			Female: 32		Severe: 26			
Vassiliadi (2021)	Greece	102	Male: 76	55	Non-ICU: 61	Survivor: 88		TSH
			Female: 26		ICU: 41	Non-survivor: 14		FT4
Vizoso (2021)	Spain	78	Male: 55	62	ICU: 78	Survivor(ICU): 55		TSH
			Female: 23			Non-survivor(ICU): 23		FT3
								FT4
Wang (2021)	China	84	Male: 53	57	Non-severe: 21	Survivor: 84	Healthy cohort: 807	TSH
			Female: 31		Severe: 63		Non-COVID-19 pneumonia: 91	
Yazan (2021)	Turkey	205	Male: 113	58	Non-ICU: 174	Survivor: 196		TSH
			Female: 92		ICU: 31	Non-survivor: 95		FT3
								FT4
Zhao (2022)	China	384	Male: 197	64	Non-severe: 161	Survivor: 219		TSH
			Female: 87		Severe: 212	Non-survivor: 16		FT3
								FT4
Zheng (2021)	China	235	Male: 112	60		Survivor: 219		TSH
			Female: 123			Non-survivor: 16		FT3
								FT4
Zou (2020)	China	149	Male: 71	49	Non-severe: 123	Survivor: 146		TSH
			Female: 78		Severe: 26	Non-survivor: 1		FT4

### Thyroxine levels at admission

A total of 36 studies recorded thyroxine levels at admission, including 5752 cases (13–24, 26, 38, 39). **eFigure 1 in the Supplement** depicts the pooled estimates of TSH (SMD = 1.46, 95% CI, [1.29, 1.62]), FT3 (SMD = 3.86, 95% CI, [3.59, 4.13]), and FT4 levels (SMD = 15.97, 95% CI, [15.24, 16.70]) by a mixed-effected model, including thirteen East Asian studies, six South Asian studies, three Asian studies, twelve European studies, one American study, and one African study. Subgroup analyses of Asian and European studies were performed (**eFigure 2 in the Supplement**). The pooled results suggested that high heterogeneity in TSH level (I^2^ = 92.9%, *p* < 0.001) and FT3 level (I^2^ = 80.3%, *p* = 0.02) but not in FT4 levels (I^2^ = 0.0%, *p*= 0.81).

### Thyroxine levels in non-COVID-19 pneumonia and healthy cohort in the COVID-19 pandemic

Six studies recorded the thyroxine levels of healthy cohorts as control groups (n = 1052). The pooled estimates of TSH, FT3, and FT4 levels were 1.78 (95% CI, [1.56, 2.01]), 4.76 (95% CI, [4.44, 5.08]), and 14.03 (95% CI, [12.93, 15.13]), respectively (**eFigure 3 in the Supplement**) (14, 18, 36, 40–42). Five studies included the thyroxine levels of non-COVID-19 pneumonia patients (n = 269) (14, 18, 42–44). The pooled estimates of TSH, FT3, and FT4 levels were 1.22 (95% CI, [1.05, 1.40[), 3.66 (95% CI, [3.48, 3.83]), and 18.19 (95% CI, [15.61, 20.78]), respectively. TSH and FT3 levels were significantly lower in patients with COVID-19 and non-COVID-19 pneumonia than in the healthy cohort (*p*< 0.001), whereas FT4 levels were significantly higher than in the healthy cohort (*p*< 0.001).

### Thyroxine levels in patients with different levels of COVID-19 severity

According to the results of single-arm meta-analysis (**eFigure 4 in the Supplement**), patients with non-severe COVID-19 (n = 1697) and severe COVID-19 (n = 1022) showed significant heterogeneity in TSH level (I^2^ = 89.9%, *P* = 0.002) and FT3 level (I^2^ = 91.9%, *P* < 0.001). Patients with non-severe COVID-19 had higher TSH and FT3 levels, and no heterogeneity in FT4 levels was found between the groups (I^2^ = 0.0%, *P* = 0.72). Ten articles compared patients with severe and non-severe COVID-19 in terms of thyroid function. Differences in the three indicators were statistically significant, and the pooled estimates of TSH, FT3, and FT4 levels were 0.40 (*P* = 0.010), 0.79 (*P* < 0.001), and −0.18 (*P* = 0.03), respectively. Three studies of the WHO classified COVID-19 severity compared patients with non-severe and severe COVID-19 (7, 28, 45). The results suggested no significant differences in TSH level (*P* = 0.54) and FT3 level (*P* = 0.06) between non-severe and severe patients, and only FT4 levels (*P* = 0.006) were significantly different (**eFigure 5 in the Supplement**), which were higher in patients with severe COVID-19. Significant heterogeneity in differences in TSH level (I^2^ = 91.0%, *P* < 0.001) and FT3 level (I^2^ = 91.0%, *P* < 0.001) was found in studies that used the WHO’s classification, whereas no significant heterogeneity was found in difference in FT4 levels (I^2^ = 0.0%, *P* = 0.39). Non-WHO analysis used an SpO2 of 93% or 94% as the cutoff between severe and non-severe COVID-19. Seven studies that used non-WHO criteria compared the thyroid functions of patients with severe and non-severe COVID-19 (13, 14, 18, 29, 43, 46, 47). The pooled estimates suggested that TSH levels were significantly lower in patients with severe COVID-19 (*P* < 0.001), and moderate heterogeneity in differences in TSH levels was found (I^2^ = 52.0%, *P* = 0.05). FT3 levels were lower in patients with severe COVID-19 (*P* < 0.001), and no significant heterogeneity in differences in FT3 levels was found (I^2^ = 0.0%, *P* =0.47). However, no difference in FT4 level was observed (*P* = 0.61). Only differences in FT4 levels were heterogeneous in the subgroup heterogeneity analysis (I^2^ = 64.9, 6%, *P* = 0.09). No significant differences in TSH level (I^2^ = 0.0%, *P* = 0.61) and FT3 level (I^2^ = 0.0%, *P* = 0.92) were found between the WHO and non-WHO clinical classifications.

### Thyroxine levels in different prognoses of patients with COVID-19

The single-arm meta-analysis (**eFigure 6 in the supplement**) showed no significant heterogeneity in FT4 levels (I^2^ = 0.0%, *P*=0.80) and significant heterogeneity in TSH level (I^2 =^ 89.4%, *P*=0.002) and FT3 level (I^2^ = 85.8%, *P*=0.008) in survivors (n = 1311) and non-survivors (n = 275). Nine articles compared the thyroxine levels of survivors and non-survivors(**eFigure 7 in the Supplement**). Differences in TSH, FT3, and FT4 levels were statistically significant, with a SMD values of 0.29 (*P* = 0.006), 1.11 (*P* < 0.001), and 0.22 (*P* < 0.001), respectively. According to the reference ranges, differences in TSH and FT4 levels were small. In the analysis of ICU patients, three studies compared survivors and non-survivors (17, 24, 32). No significant difference in TSH (*P* = 0.74) levels was found among ICU patients, but significant difference in FT4 (*P* = 0.003) and FT3 (*P* = 0.001) levels were found. The survivors had significantly higher FT4 and FT3 levels than non-survivors. The pooled mean differences in FT3 and FT4 levels were 0.51 (95% CI, [0.21, 0.82]) and 0.47 (95% CI, [0.16, 0.77]). Six studies included COVID-19 patients in all wards (13, 19, 24, 29, 35, 48). Significant differences in TSH (*P* < 0.001), FT3 (*P* < 0.001) and FT4 (*P* = 0.02) levels were found, with mean differences of 0.46 (95% CI,[0.25,0.67]), 1.39 (95% CI,[0.86,1.92]), and 0.17 (95% (49) CI,[0.03.0.31]), respectively. All three indexes were higher in survivors.

### Thyroxine levels during follow-up

Seven studies reassessed thyroid function after acute COVID-19 (**eFigure 8 in the Supplement**) (17, 18, 27, 34, 41, 43, 50). The pooled estimates of TSH, FT3, and FT4 levels were 1.53 (95% CI, [1.36, 1.70]), 4.74 (95% CI, [4.36, 5.11]), and 14.07 (95% CI, [11.90, 16.25]), respectively, without significant heterogeneity across studies. TSH, FT3, and FT4 levels were recovered during follow-up compared with the acute period. TSH and FT3 levels increased, whereas FT4 levels decreased compared with the acute phase.

## Discussion

SARS-CoV-2 can damage multiple organs, including the lungs, liver, heart, brain, and kidneys, leading to systemic symptoms. The thyroid gland highly expresses the ACE2 receptor. Thus, the hypothalamic–pituitary–thyroid axis may be susceptible to the disturbance in patients with COVID19 (22, 34, 41, 51, 52). SARS-CoV, a coronavirus related to SARS-CoV-2, injures thyroid parafollicular and follicular cells (53). Lui et al. found that high SARS-CoV-2 viral loads were associated with small thyroid volumes (37). This association suggested a direct viral effect on the thyroid gland. Lania et al. revealed that COVID-19 might be associated with the high risk of thyrotoxicosis (n = 31, 10.8%) in a retrospective study that enrolled 287 patients (54). However, the autopsy results suggested the absence of the virus was in thyroid tissues (55–57). The number of reported thyrotoxicosis cases in literature did not exponentially increase, including critically ill patients (13, 15, 28, 32, 34). Thyrotoxicosis may be a rare complication of COVID-19 (51).

Many studies revealed that thyroid function significantly changes during COVID-19 infection. The trends of thyroxine level fluctuations in patients with COVID-19 and non-COVID-19 pneumonia were similar (decreased TSH and FT3 levels and increased FT4 levels). This similarity suggests that COVID-19 and non-COVID-19 pneumonia affect the thyroid gland through similar mechanisms. In addition, thyroxine levels during follow-up indicated progressive improvement and transient hormone changes (17, 34). Patients suffering from COVID-19 and thyroxine fluctuation potentially encountered non-thyroidal illness syndrome (NTIS) induced by systemic inflammation (58, 59). NTIS is an adaptive response to stress, critical illness, and malnutrition, manifested by a decrease in FT3 levels or decreases in TSH, FT3, and FT4 levels in severe disease (58, 60). Since systemic inflammation potentially impacts the de-iodinase activity, it inhibits T4–T3 conversion decreases FT3 levels and increases FT4 levels (17, 61, 62). Elevated FT4 level upon admission tends to be mistaken for thyrotoxicosis. Most studies have investigated thyroxine changes in COVID-19 at admission. NTIS in patients with long-term critical illness shows symptoms similar to hypothyroidism (59, 63, 64). Unlike thyrotoxicosis, treatment with thyroid hormone is not recommended without clinical signs of hypothyroidism. Even mild hypothyroidism can be considered a physiologically favorable condition that can suppress energy expenditure and eventually restrict catabolism by decreasing thyroid hormone activity (63). Thyroid function can recover in patients without intervention (18, 58, 65, 66).

Immunoassays for thyroxine can be affected by alterations in serum binding protein that occurs in various physiological states (20). Decreased TSH and increased FT4 levels were reported in the healthy cohort during the COVID-19 outbreak (67). During the pandemic, relationships were found between thyroid diseases and psychiatric factors, such as anxiety and depression (68, 69). The pandemic socio-psychological sequelae can constitute stressors for the population, potentially affecting the thyroid gland. Collectively, patients with COVID-19 are at risk of thyroid diseases and require attention.

Thyroid function parameters have clinical significance in determining disease severity and prognosis of COVID-19. Whether the severity of COVID-19 is associated with thyroid function remains unclear. Some studies reported no significant relationship between COVID-19 severity and thyroid function, whereas other studies have suggested that only some statistically significant indicators compare disease severity (7, 13, 14, 28, 45, 47). It may be related to multiple factors, including different criteria adopted in different regions. The classifications of COVID-19 severity vary. The WHO uses SpO2 of less than 90% as a cutoff between severe and non-severe COVID-19. By contrast, the United States, China, and Japan use 93% or 94% as a division basis in diagnosis and treatment guidelines (14, 19, 47, 70). In the present study, the single-arm meta-analysis results revealed that only TSH and FT3 levels were significantly different compared to patients with severe-critical and non-severe COVID-19. The case-control study showed that although the FT4 levels differed significantly, the difference was only 0.18, with minor practicality. In the acute phase, TSH and FT3 levels at admission in COVID-19 patients can be used in assessing patient severity.

Thyroid function is not a routine test indicator in patients infected with COVID-19. Some studies have suggested that thyroid function cannot be used in evaluating the prognosis of patients, or only some indicators can be used in the prognostic analysis (13, 32, 48, 71). In the present study, TSH, FT3, and FT4 levels presented significant differences between survivors and deceased patients at admission. The results of the single-arm meta-analysis suggested that only TSH and FT3 levels were heterogeneous between survivors and non-survivors. The controlled studies showed statistically significant differences in the three thyroid function parameters. However, we found that differences in TSH (SMD = 0.29) and FT4 (SMD = 0.22) levels were limited for assessing prognosis. Compared with TSH and FT4, FT3 levels may be more effective (SMD = 0.79). A low FT3 level is an outcome predictor, especially in severe patients (5). The excessive production of proinflammatory cytokines during SARS-CoV-2 infection aggravates ARDS and tissue damage resulting in multi-organ failure and death (72, 73). In patients with COVID-19, FT3 levels decrease with the increasing levels of inflammatory cytokines. Some studies have suggested that improving thyroid function can improve patient outcomes. However, a reduction in FT3 levels occurs before clinical symptoms worsen. This reduction can be used in assessing changes in patient condition and prognosis (74).

Survivor bias may have been present when prognostic thyroid function was being determined. Thyroid function is an unconventional test indicator for patients with COVID-19. Medical centers tend to assess the thyroid function in patients with underlying thyroid diseases or other severe illnesses. Moreover, the pharmacological doses of steroids in severe COVID-19 can affect thyroid function. Therefore, comprehensive extensive clinical studies are needed to evaluate the significance of thyroid function assessment for patients with COVID-19.

### Limitations

This study has several limitations. First, thyroid function is not a routine test indicator for COVID-19. Many studies have a selection bias for patients tested for thyroid function levels and have an incomplete recording of test results, especially TT3 and TT4 results. Second, heterogeneity was found among the included studies. Third, thyroid function often showed non-normal distribution. The median percentage transformation was used in the meta-analysis. Fourth, the cohort studies’ sample size with a detailed thyroxine record was limited after reasonable sorting.

## Conclusion

Thyroxine levels (TSH, FT3, and FT4) fluctuated in patients with COVID-19. Compared with the healthy cohort, patients with COVID-19 showed decreased TSH and FT3 levels and increased FT4 levels. There were differences in thyroxine levels between severe and non-severe patients. No significant difference in TSH levels between severe and non-severe patients according to the WHO classification criteria, whereas FT4 levels were not significantly different in the studies using non-WHO classification criteria. However, FT3 levels were significantly lower in severe patients than in non-severe patients in the included study. In addition, different thyroid function parameters were assessed differently with regard to patient outcomes. TSH and FT4 levels have limitations in the prognostic evaluation of ICU patients and are ineffective in assessing patient outcomes. In general wards, TSH and FT4 are still effective, but the clinical application value is limited due to slight differences. FT3 levels can be adapted as an outcome assessment indicator for patients in ICU or not, with a reliable scope of application. Following the resolution of COVID-19 pneumonia, thyroid function gradually recovers in survivors during follow-up.

## Data availability statement

The original contributions presented in the study are included in the article/**Supplementary Material**. Further inquiries can be directed to the corresponding authors.

## Ethics statement

This is a meta-analysis based on observational studies. Informed consent from patients and ethical approval for this type of study are not required.

## Author contributions

ZL and PH had full access to all of the data in the study and take responsibility for the integrity of the data and the accuracy of the data analysis. ZL and PH contributed equally. Concept and design: ZL, PH, HM, SM, MF, SW, YF. Acquisition, analysis, or interpretation of data: ZL, PH, SM, MF, HW, WZ, YC, SW, YF. Drafting of the manuscript: ZL, PH, SW, YF. Critical revision of the manuscript for important intellectual content: all authors. Statistical analysis: ZL, SW, YF. Administrative, technical, or material support: SM, MF, HW, WZ. Supervision: HM, SM, MF, HW, WZ, YC, PH, SW, YF. Other - protocol review: ZL. All authors contributed to the article and approved the submitted version.

## References

[1] Sohrabi C Alsafi Z O'Neill N Khan M Kerwan A Al-Jabir A. World Health Organization declares global emergency: A review of the 2019 novel coronavirus (COVID-19). Int J Surg. (2020) 76:71–76. doi: 10.1016/j.ijsu.2020.02.034 PMC7105032 32112977

[2] Horton R. Offline: 2019-nCoV outbreak-early lessons. Lancet (2020) 395:322. doi: 10.1016/S0140-6736(20)30212-9 32007152 PMC7137834

[3] Huang C Wang Y Li X Ren L Zhao J Hu Y. Clinical features of patients infected with 2019 novel coronavirus in wuhan, China. Lancet (2020) 395:497–506. doi: 10.1016/S0140-6736(20)30183-5 31986264 PMC7159299

[4] Chen N Zhou M Dong X Qu J Gong F Han Y. Epidemiological and clinical characteristics of 99 cases of 2019 novel coronavirus pneumonia in wuhan, China: a descriptive study. Lancet (2020) 395:507–13. doi: 10.1016/S0140-6736(20)30211-7 PMC7135076 32007143

[5] Llamas M Garo ML Giovanella L. Low free-T3 serum levels and prognosis of COVID-19: systematic review and meta-analysis. Clin Chem Lab Med (2021) 59:1906–13. doi: 10.1515/cclm-2021-0805 34380183

[6] Dworakowska D Grossman AB. Thyroid disease in the time of COVID-19. Endocrine (2020) 68:471–4. doi: 10.1007/s12020-020-02364-8 PMC7275975 32507963

[7] Kumar B Gopalakrishnan M Garg MK Purohit P Banerjee M Sharma P. Endocrine dysfunction among patients with COVID-19: A single-center experience from a tertiary hospital in India. Indian J Endocrinol Metab (2021) 25:14–9. doi: 10.4103/ijem.IJEM_577_20 PMC8323627 34386388

[8] van Gerwen M Alsen M Little C Barlow J Naymagon L Tremblay D. Outcomes of patients with hypothyroidism and COVID-19: A retrospective cohort study. Front Endocrinol (Lausanne) (2020) 11:565–70. doi: 10.3389/fendo.2020.00565 PMC7461836 33013686

[9] Trimboli P Camponovo C Scappaticcio L Bellastella G Piccardo A Rotondi M. Thyroid sequelae of COVID-19: a systematic review of reviews. Rev Endocr Metab Disord (2021) 22:485–91. doi: 10.1007/s11154-021-09653-1 PMC8038866 33843008

[10] Lisco G De Tullio A Jirillo E Giagulli VA De Pergola G. Guastamacchia e and triggiani v. thyroid and COVID-19: a review on pathophysiological, clinical and organizational aspects. J Endocrinol Invest (2021) 44:1801–14. doi: 10.1007/s40618-021-01554-z PMC7992516 33765288

[11] Hariyanto TI Kurniawan A. Thyroid disease is associated with severe coronavirus disease 2019 (COVID-19) infection. Diabetes Metab Syndr (2020) 14:1429–30. doi: 10.1016/j.dsx.2020.07.044 PMC7387272 32755846

[12] Wan X Wang W Liu J Tong T. Estimating the sample mean and standard deviation from the sample size, median, range and/or interquartile range. BMC Med Res Methodol (2014) 14:135–48. doi: 10.1186/1471-2288-14-135 PMC4383202 25524443

[13] Ahn J Lee MK Lee JH Sohn SY. Thyroid hormone profile and its prognostic impact on the coronavirus disease 2019 in Korean patients. Endocrinol Metab (Seoul) (2021) 36:769–77. doi: 10.3803/EnM.2021.1109 PMC8419615 34474515

[14] Assimakopoulos SF Markantes GK Papageorgiou D Mamali I Markou KB Marangos M. Low serum TSH in the acute phase of COVID-19 pneumonia: thyrotoxicosis or a face of "non-thyroidal illness syndrome"? Clin Chem Lab Med (2021) 59:e420–3. doi: 10.1515/cclm-2021-0511 34246200

[15] Baldelli R Nicastri E Petrosillo N Marchioni L Gubbiotti A Sperduti I. Thyroid dysfunction in COVID-19 patients. J Endocrinol Invest (2021) 44:2735–9. doi: 10.1007/s40618-021-01599-0 PMC8185485 34101132

[16] Beltrao FEL Beltrao DCA Carvalhal G Beltrao FEL Brito ADS Capistrano K. Thyroid hormone levels during hospital admission inform disease severity and mortality in COVID-19 patients. Thyroid (2021) 31:1639–49. doi: 10.1089/thy.2021.0225 34314259

[17] Campi I Bulgarelli I Dubini A Perego GB Tortorici E Torlasco C. The spectrum of thyroid function tests during hospitalization for SARS COV-2 infection. Eur J Endocrinol (2021) 184:699–709. doi: 10.1530/EJE-20-1391 33683214 PMC9494333

[18] Chen M Zhou W Xu W. Thyroid function analysis in 50 patients with COVID-19: A retrospective study. Thyroid (2021) 31:8–11. doi: 10.1089/thy.2020.0363 32600165

[19] Chen T Wu D Chen H Yan W Yang D Chen G. Clinical characteristics of 113 deceased patients with coronavirus disease 2019: retrospective study. BMJ (2020) 368:1091–105. doi: 10.1136/bmj.m1091 PMC7190011 32217556

[20] Clausen CL Rasmussen ÅK Johannsen TH Hilsted LM Skakkebæk NE Szecsi PB. Thyroid function in COVID-19 and the association with cytokine levels and mortality. Endocr Connect (2021) 10:1234–42. doi: 10.1530/EC-21-0301 PMC8494417 34468398

[21] Dabas A Singh H Goswami B Kumar K Dubey A Jhamb U. Thyroid dysfunction in COVID-19. Indian J Endocrinol Metab (2021) 25:198–201. doi: 10.4103/ijem.ijem_195_21 34760673 PMC8547402

[22] D'Ardes D Rossi I Bucciarelli B Allegra M Bianco F Sinjari B. Metabolic changes in SARS-CoV-2 infection: Clinical data and molecular hypothesis to explain alterations of lipid profile and thyroid function observed in COVID-19 patients. Life (Basel) (2021) 11:860–9. doi: 10.3390/life11080860 PMC8400261 34440605

[23] Das L Dutta P Walia R Mukherjee S Suri V Puri GD. Spectrum of endocrine dysfunction and association with disease severity in patients with COVID-19: Insights from a cross-sectional, observational study. Front Endocrinol (Lausanne) (2021) 12:645787. doi: 10.3389/fendo.2021.645787 34276556 PMC8283965

[24] Dincer Yazan C Ilgin C Elbasan O Apaydin T Dashdamirova S Yigit T. The association of thyroid hormone changes with inflammatory status and prognosis in COVID-19. Int J Endocrinol (2021) 2021:2395212. doi: 10.1155/2021/2395212 34422043 PMC8371668

[25] Kumar B Gopalakrishnan M Garg MK Purohit P Banerjee M Sharma P. Endocrine dysfunction among patients with COVID-19: A s@ingle-center experience from a tertiary hospital in India. Indian J Endocrinol Metab (2021) 25:14–9. doi: 10.4103/ijem.IJEM_577_20 PMC8323627 34386388

[26] Ballesteros Vizoso MA Castilla AF Barcelo A Raurich JM Argente Del Castillo P Morell-Garcia D. Thyroid disfunction in critically ill COVID-19 patients. relationship with in-hospital mortality. J Clin Med (2021) 10:5057–64. doi: 10.3390/jcm10215057 PMC8584356 34768580

[27] Clarke SA Phylactou M Patel B Mills EG Muzi B Izzi-Engbeaya C. Normal adrenal and thyroid function in patients who survive COVID-19 infection. J Clin Endocrinol Metab (2021) 106:2208–20. doi: 10.1210/clinem/dgab349 PMC8194556 34008009

[28] Dutta A Jevalikar G Sharma R Farooqui KJ Mahendru S Dewan A. Low FT3 is an independent marker of disease severity in patients hospitalized for COVID-19. Endocr Connect (2021) 10:1455–62. doi: 10.1530/EC-21-0362 PMC8630756 34662295

[29] Gao W Guo W Guo Y Shi M Dong G Wang G. Thyroid hormone concentrations in severely or critically ill patients with COVID-19. J Endocrinol Invest (2021) 44:1031–40. doi: 10.1007/s40618-020-01460-w PMC7605732 33140379

[30] Gong J Wang DK Dong H Xia QS Huang ZY Zhao Y. Prognostic significance of low TSH concentration in patients with COVID-19 presenting with non-thyroidal illness syndrome. BMC Endocr Disord (2021) 21:111. doi: 10.1186/s12902-021-00766-x 34044831 PMC8159017

[31] Grondman I de Nooijer AH Antonakos N Janssen NAF Mouktaroudi M Leventogiannis K. The association of TSH and thyroid hormones with lymphopenia in bacterial sepsis and COVID-19. J Clin Endocrinol Metab (2021) 106:1994–2009. doi: 10.1210/clinem/dgab148 33713408 PMC7989224

[32] Guven M Gultekin H. The prognostic impact of thyroid disorders on the clinical severity of COVID-19: Results of single-centre pandemic hospital. Int J Clin Pract (2021) 75:e14129. doi: 10.1111/ijcp.14129 33655591 PMC7995023

[33] Zhao H Dang W Zhou L Xiong J Li Y. The prognostic value of free triiodothyronine for COVID-19 patients. J Inter Intensive Med (2022) 28:32–6. doi: 10.11768/nkjwzzzz20220107

[34] Khoo B Tan T Clarke SA Mills EG Patel B Modi M. Thyroid function before, during, and after COVID-19. J Clin Endocrinol Metab (2021) 106:e803–11. doi: 10.1210/clinem/dgaa830 PMC7823247 33180932

[35] Lang S Liu Y Qu X Lu R Fu W Zhang W. Association between thyroid function and prognosis of COVID-19: A retrospective observational study. Endocr Res (2021) 46:170–7. doi: 10.1080/07435800.2021.1924770 34014139

[36] Li T Wang L Wang H Gao Y Hu X Li X. Characteristics of laboratory indexes in COVID-19 patients with non-severe symptoms in hefei city, China: Diagnostic value in organ injuries. Eur J Clin Microbiol Infect Dis (2020) 39:2447–55. doi: 10.1007/s10096-020-03967-9 PMC7329266 32613308

[37] Lui DTW Fung MMH Chiu KWH Lee CH Chow WS Lee ACH. Higher SARS-CoV-2 viral loads correlated with smaller thyroid volumes on ultrasound among male COVID-19 survivors. Endocrine (2021) 74:205–14. doi: 10.1007/s12020-021-02855-2 PMC8408037 34467467

[38] Lui DTW Lee CH Chow WS Lee ACH Tam AR Pang P. The independent association of TSH and free triiodothyronine levels with lymphocyte counts among COVID-19 patients. Front Endocrinol (Lausanne) 2021 12:774346. doi: 10.3389/fendo.2021.774346 35095756 PMC8792436

[39] Kumar B Gopalakrishnan M Garg MK Purohit P Banerjee M Sharma P. Endocrine dysfunction among PatPients with COVID-19: A single-center experience from a tertiary hospital in India. Indian J Endocrinol Metab (2021) 25:14–9. doi: 10.4103/ijem.IJEM_577_20 PMC8323627 34386388

[40] Okwor CJ Meka IA Akinwande KS Edem VF Okwor VC. Assessment of thyroid function of newly diagnosed SARS-CoV-2 infected patients in Nigeria. Pan Afr Med J (2021) 40:9–18. doi: 10.11604/pamj.2021.40.9.26358 PMC8490165 34650659

[41] Urhan E Karaca Z Kara CS Yuce ZT Unluhizarci K. The potential impact of COVID-19 on thyroid gland volumes among COVID-19 survivors. Endocrine (2022) 76:635–41. doi: 10.1007/s12020-022-03019-6 PMC8892112 35239124

[42] Wang W Su X Ding Y Fan W Zhou W Su J. Thyroid function abnormalities in COVID-19 patients. Front Endocrinol (Lausanne) (2020) 11:623792. doi: 10.3389/fendo.2020.623792 33679608 PMC7933556

[43] Malik J Malik A Javaid M Zahid T Ishaq U Shoaib M. Thyroid function analysis in COVID-19: A retrospective study from a single center. PLos One (2021) 16:e0249421. doi: 10.1371/journal.pone.0249421 33784355 PMC8009384

[44] Okoye C Niccolai F Rogani S Lemmi B Peta U Del Vecchio S. Is non-thyroidal illness syndrome (NTIS) a clinical predictor of COVID-19 mortality in critically ill oldest old patients? J Endocrinol Invest (2022), 45(9):1689–92. doi: 10.1007/s40618-022-01806-6 PMC9094134 35545741

[45] Sen K Sinha A Sen S Chakraborty S Alam MS. Thyroid function test in COVID-19 patients: A cross-sectional study in a tertiary care hospital. Indian J Endocrinol Metab (2020) 24:532–6. doi: 10.4103/ijem.IJEM_779_20 PMC7906107 33643870

[46] Lui DTW Lee CH Chow WS Lee ACH Tam AR Fong CHY. Thyroid dysfunction in relation to immune profile, disease status, and outcome in 191 patients with COVID-19. J Clin Endocrinol Metab (2021) 106:e926–35. doi: 10.1210/clinem/dgaa813 PMC7665541 33141191

[47] Nakamura S Kido N Watanabe M Ohmachi Y Inayama Y Kashitani Y. Analysis of thyroid function in Japanese patients with coronavirus disease 2019. Endocr J (2022) 69:643–8. doi: 10.1507/endocrj.EJ21-0609 34955465

[48] Schwarz Y Percik R Oberman B Yaffe D Zimlichman E Tirosh A. Sick euthyroid syndrome on presentation of patients with COVID-19: A potential marker for disease severity. Endocr Pract (2021) 27:101–9. doi: 10.1016/j.eprac.2021.01.001 PMC7834506 33551316

[49] Knief U Forstmeier W. Violating the normality assumption may be the lesser of two evils. Behav Res Methods (2021) 53:2576–90. doi: 10.3758/s13428-021-01587-5 PMC8613103 33963496

[50] Lui DTW Lee CH Chow WS Lee ACH Tam AR Pang P. Long COVID in patients with mild to moderate disease: Do thyroid function and autoimmunity play a role? Endocr Pract (2021) 27:894–902. doi: 10.1016/j.eprac.2021.06.016 34237471 PMC8257401

[51] Trimboli P Cappelli C Croce L Scappaticcio L Chiovato L Rotondi M. COVID-19-Associated subacute thyroiditis: Evidence-based data from a systematic review. Front Endocrinol (Lausanne) (2021) 12:707726. doi: 10.3389/fendo.2021.707726 34659109 PMC8511511

[52] Hashemipour S Shahsavari P Kiani S Badri M Ghobadi A Hadizadeh Khairkhahan SMR. Wide spectrum of thyroid function tests in COVID-19: From nonthyroidal illness to isolated hyperthyroxinemia. Int J Endocrinol Metab (2022) 20:e120709. doi: 10.5812/ijem.120709 35432556 PMC8994829

[53] Wei L Sun S Xu CH Zhang J Xu Y Zhu H. Pathology of the thyroid in severe acute respiratory syndrome. Hum Pathol (2007) 38:95–102. doi: 10.1016/j.humpath.2006.06.011 16996569 PMC7112059

[54] Lania A Sandri MT Cellini M Mirani M Lavezzi E Mazziotti G. Thyrotoxicosis in patients with COVID-19: the THYRCOV study. Eur J Endocrinol (2020) 183:381–7. doi: 10.1530/EJE-20-0335 PMC9494315 32698147

[55] Bradley BT Maioli H Johnston R Chaudhry I Fink SL Xu H. Histopathology and ultrastructural findings of fatal COVID-19 infections in Washington state: A case series. Lancet (2020) 396:320–32. doi: 10.1016/S0140-6736(20)31305-2 PMC7365650 32682491

[56] Barton LM Duval EJ Stroberg E Ghosh S and Mukhopadhyay S. COVID-19 autopsies, Oklahoma, USA. Am J Clin Pathol (2020) 153:725–33. doi: 10.1093/ajcp/aqaa062 PMC7184436 32275742

[57] Yao X Li T Li Z Ping Y Liu H Yu S. A pathological report of three COVID-19 cases by minimal invasive autopsies. Chin J Pathol (2020) 49:411–7. doi: 10.3760/cma.j.cn112151-20200312-00193 32172546

[58] Sciacchitano S Capalbo C Napoli C Anibaldi P Salvati V De Vitis C. Nonthyroidal illness syndrome: To treat or not to treat? have we answered the question? a review of metanalyses. Front Endocrinol (Lausanne) (2022) 13:850328. doi: 10.3389/fendo.2022.850328 35620389 PMC9128382

[59] Bello G Pennisi MA Montini L Silva S Maviglia R Cavallaro F. Nonthyroidal illness syndrome and prolonged mechanical ventilation in patients admitted to the ICU. Chest (2009) 135:1448–54. doi: 10.1378/chest.08-1816 19255297

[60] Zou R Wu C Zhang S Wang G Zhang Q Yu B. Euthyroid sick syndrome in patients with COVID-19. Front Endocrinol (Lausanne) (2020) 11:566439. doi: 10.3389/fendo.2020.566439 33117282 PMC7575767

[61] Ilera V Delfino LC Zunino A Glikman P Drnovsek M Reyes A. Correlation between inflammatory parameters and pituitary-thyroid axis in patients with COVID-19. Endocrine (2021) 74:455–60. doi: 10.1007/s12020-021-02863-2 PMC8436010 34515958

[62] Scappaticcio L Pitoia F Esposito K Piccardo A Trimboli P. Impact of COVID-19 on the thyroid gland: An update. Rev Endocr Metab Disord (2021) 22:803–15. doi: 10.1007/s11154-020-09615-z PMC7688298 33241508

[63] Fliers E Bianco AC Langouche L Boelen A. Thyroid function in critically ill patients. Lancet Diabetes Endocrinol (2015) 3:816–25. doi: 10.1016/S2213-8587(15)00225-9 PMC4979220 26071885

[64] Arem R Wiener GJ Kaplan SG Kim HS Reichlin S Kaplan MM. Reduced tissue thyroid hormone levels in fatal illness. Metabolism (1993) 42:1102–8. doi: 10.1016/0026-0495(93)90266-Q 8412761

[65] Bartalena L. The dilemma of non-thyroidal illness syndrome: To treat or not to treat? J Endocrinol Invest (2003) 26:1162. doi: 10.1007/BF03349150 15055465

[66] Peeters RP. Non thyroidal illness: to treat or not to treat? Ann Endocrinol (Paris) (2007) 68:224–8. doi: 10.1016/j.ando.2007.06.011 17689479

[67] Weiwei D Bei W Hong W Cailan W Hailin S Donghong X. Thyroid hormone changes in the northern area of tianjin during the COVID-19 pandemic. Int J Endocrinol (2022) 2022:5720875. doi: 10.1155/2022/5720875 35013681 PMC8742148

[68] Siegmann EM Müller HHO Luecke C Philipsen A Kornhuber J Grömer TW. Association of depression and anxiety disorders with autoimmune thyroiditis: A systematic review and meta-analysis. JAMA Psychiatry (2018) 75:577–84. doi: 10.1001/jamapsychiatry.2018.0190 PMC6137529 29800939

[69] Fischer S Ehlert U. Hypothalamic-pituitary-thyroid (HPT) axis functioning in anxiety disorders. a systematic review. Depress Anxiety (2018) 35:98–110. doi: 10.1002/da.22692 29064607

[70] Wang C An X Li S Zheng H Chen Y Du B. Comparision of guidelines on coronavirus disease 2019 between countries and world health organization. Chin J Crit Care Intensive Care Med (2020) 6:383–92. doi: 10.3877/cma.j.issn.2096-1537.2020.04.007

[71] Goyal A Gupta Y Kalaivani M Tandon N. Mild and asymptomatic SARS-CoV-2 infection is not associated with progression of thyroid dysfunction or thyroid autoimmunity. Clin Endocrinol (Oxf) (2022) 98(2):277–79. doi: 10.1111/cen.14731 PMC9111349 35384011

[72] Coperchini F Chiovato L Croce L Magri F Rotondi M. The cytokine storm in COVID-19: An overview of the involvement of the chemokine/chemokine-receptor system. Cytokine Growth Factor Rev (2020) 53:25–32. doi: 10.1016/j.cytogfr.2020.05.003 32446778 PMC7211650

[73] Zhang YY Li BR Ning BT. The comparative immunological characteristics of SARS-CoV, MERS-CoV, and SARS-CoV-2 coronavirus infections. Front Immunol (2020) 11:2033. doi: 10.3389/fimmu.2020.02033 32922406 PMC7457039

[74] Ragab D Salah Eldin H Taeimah M Khattab R Salem R. The COVID-19 cytokine storm; what we know so far. Front Immunol (2020) 11:1446. doi: 10.3389/fimmu.2020.01446 32612617 PMC7308649

