# Supplementary material for: Thyroxine changes in COVID-19 pandemic: A systematic review and meta-analysis
Source: Front Endocrinol (Lausanne). 2023 Feb 13;14:1089190. doi: 10.3389/fendo.2023.1089190 (PMC9969987; doi:10.3389/fendo.2023.1089190)

**Supplemental Online Content**

**eMethods 1.** MOOSE Checklist

**eTable 1.** Quality scores of included studies using newcastle-ottawa scale.

**eTable 2.** Modified Newcastle-Ottawa Quality Assessment Scale

**eFigure 1.** Forest plot for all studies comparing the Thyroxine levels at admission.

**eFigure 2.** Forest plot comparing the Thyroxine levels between Asian and European studies.

**eFigure 3.** Forest plot comparing the Thyroxine levels between non-COVID-19 pneumonia and healthy cohort in the COVID-19 pandemic.

**eFigure 4.** Forest plot for all studies comparing the Thyroxine levels in patients with different levels of COVID-19 severity.

**eFigure 5.** Forest plot comparing the different clinical classification of Thyroxine levels between the severe patients and nonsevere patients with COVID-19.

**eFigure 6.** Forest plot for all studies comparing Thyroxine levels in different prognoses of patients with COVID-19.

**eFigure 7.** Forest plot comparing the Thyroxine levels in different prognoses between ICU and All ward patients with COVID-19.

**eFigure 8.** Forest plot comparing the Thyroxine levels during follow-up.

| **Item No**  **eMethods 1. MOOSE** Checklist | **Recommendation** | **Reported on Page No** |
| --- | --- | --- |
| Reporting of background should include | | |
| 1 | Problem definition | 2 |
| 2 | Hypothesis statement | 2 |
| 3 | Description of study outcome(s) | 3 |
| 4 | Type of exposure or intervention used | 5 |
| 5 | Type of study designs used | 5 |
| 6 | Study population | 5 |
| Reporting of search strategy should include | | |
| 7 | Qualifications of searchers (eg, librarians and investigators) | None |
| 8 | Search strategy, including time period included in the synthesis and key words | 5 |
| 9 | Effort to include all available studies, including contact with authors | 5 |
| 10 | Databases and registries searched | 6 |
| 11 | Search software used, name and version, including special features used (eg, explosion) | 7 |
| 12 | Use of hand searching (eg, reference lists of obtained articles) | 5 |
| 13 | List of citations located and those excluded, including justification | 6 |
| 14 | Method of addressing articles published in languages other than English | 6 |
| 15 | Method of handling abstracts and unpublished studies | 6 |
| 16 | Description of any contact with authors | 6 |
| Reporting of methods should include | | |
| 17 | Description of relevance or appropriateness of studies assembled for assessing the hypothesis to be tested | 5 |
| 18 | Rationale for the selection and coding of data (eg, sound clinical principles or convenience) | 6 |
| 19 | Documentation of how data were classified and coded (eg, multiple raters, blinding and interrater reliability) | 6 |
| 20 | Assessment of confounding (eg, comparability of cases and controls in studies where appropriate) | 6 |
| 21 | Assessment of study quality, including blinding of quality assessors, stratification or regression on possible predictors of study results | 7 |
| 22 | Assessment of heterogeneity | 7 |
| 23 | Description of statistical methods (eg, complete description of fixed or random effects models, justification of whether the chosen models account for predictors of study results, dose-response models, or cumulative meta-analysis) in sufficient detail to be replicated | 7 |
| 24 | Provision of appropriate tables and graphics | 7 |
| Reporting of results should include | | |
| 25 | Graphic summarizing individual study estimates and overall estimate | 8 |
| 26 | Table giving descriptive information for each study included | 8 |
| 27 | Results of sensitivity testing (eg, subgroup analysis) | 8 |
| 28 | Indication of statistical uncertainty of findings | None |

**eTable 1. Quality scores of included studies using newcastle-ottawa scale.**

**No.**

1

2

3

4

5

6

7

8

9

10

11

12

13

14

15

16

17

18

19

20

21

22

23

**First author**

[Ahn](https://pubmed.ncbi.nlm.nih.gov/?size=50&amp;term=Ahn%2BJ&amp;cauthor_id=34474515) Ardes Baldelli Beltrão [Campi](https://pubmed.ncbi.nlm.nih.gov/?size=50&amp;term=Campi%2BI&amp;cauthor_id=33683214)

[Chen](https://pubmed.ncbi.nlm.nih.gov/?size=50&amp;term=Chen%2BT&amp;cauthor_id=32217556) [Chen](https://pubmed.ncbi.nlm.nih.gov/?size=50&amp;term=Chen%2BM&amp;cauthor_id=32600165) Clarke Clausen Dabas Das [Dutta](https://pubmed.ncbi.nlm.nih.gov/?size=50&amp;term=Dutta%2BA&amp;cauthor_id=34662295) [Gao](https://pubmed.ncbi.nlm.nih.gov/?size=50&amp;term=Gao%2BW&amp;cauthor_id=33140379) Gong

[Grondman](https://pubmed.ncbi.nlm.nih.gov/?size=50&amp;term=Grondman%2BI&amp;cauthor_id=33713408) [Güven](https://pubmed.ncbi.nlm.nih.gov/?size=50&amp;term=G%C3%BCven%2BM&amp;cauthor_id=33655591) Khoo Kumar [Lang](https://pubmed.ncbi.nlm.nih.gov/?size=50&amp;term=Lang%2BS&amp;cauthor_id=34014139)

LI

Lui [Malik](https://pubmed.ncbi.nlm.nih.gov/?size=50&amp;term=Malik%2BJ&amp;cauthor_id=33784355)

Nakamura

**Year**

2021

2021

2021

2021

2021

2019

2020

2021

2021

2021

2021

2021

2020

2021

2021

2020

2021

2021

2021

2020

2020-2021

2021

2021

**Selection**

****

****

****

***

****

****

****

****

****

****

****

****

****

***

****

****

****

****

***

****

****

***

***

**Comparability**

**

**

**

**

**

**

**

**

**

**

**

**

**

**

**

**

**

*

*

**

**

**

*

**Outcome**

**

***

***

***

***

***

***

***

**

**

***

***

***

***

***

**

***

**

**

**

***

***

***

**Nos**

8*

9*

9*

9*

9*

9*

9*

9*

8*

8*

9*

9*

9*

8*

9*

8*

9*

7*

6*

8*

9*

8*

7*

1. [Okoye](https://pubmed.ncbi.nlm.nih.gov/?size=50&amp;term=Okoye%2BC&amp;cauthor_id=35545741)
2. Okwor
3. Schwarz
4. [Sciacchitano](https://pubmed.ncbi.nlm.nih.gov/?size=50&amp;term=Sciacchitano%2BS&amp;cauthor_id=34861865)
5. Sen
6. Sparano
7. Urhan
8. Vassiliadi
9. [Vizoso](https://pubmed.ncbi.nlm.nih.gov/?size=50&amp;term=Ballesteros%2BVizoso%2BMA&amp;cauthor_id=34768580)
10. Wang
11. [Yazan](https://pubmed.ncbi.nlm.nih.gov/?size=50&amp;term=Dincer%2BYazan%2BC&amp;cauthor_id=34422043)
12. Zhao
13. [Zheng](https://pubmed.ncbi.nlm.nih.gov/?size=50&amp;term=Zheng%2BJ&amp;cauthor_id=34781943)
14. Zou

2022

2021

2021

2021

2020

2021

2021

2021

2021

2021

2021

2021

2021

2020

**** **

**** **

*** **

**** **

**** **

**** *

*** **

**** **

**** **

**** **

**** **

**** *

**** **

**** **

*** 9*

** 8*

*** 8*

*** 9*

** 8*

** 7*

*** 8*

** 8*

*** 9*

*** 9*

*** 9*

*** 8*

*** 9*

*** 9*

Selection:

* :Meet the one item in the NOS selection section.

** :Meet the two items in the NOS selection section.

*** : Meet the three items in the NOS selection section.

**** :Meet the four items in the NOS selection section. Comparability:

- :The comparability of the study cohort design was of medium quality.

** :The comparability of the study cohort design was of high quality. Outcome:

- :Meet the one item in the NOS outcome section.

** :Meet the two items in the NOS outcome section.

*** : Meet the three items in the NOS outcome section.

**eTable 2. Modified Newcastle-Ottawa Quality Assessment Scale**

| **Assessment of quality of a cohort study – Newcastle Ottawa Scale** | | |
| --- | --- | --- |
| **Selection** (tick one box in each section) | | |
| 1. Representativeness of the intervention cohort  a) Truly representative of the patients with COVID-19  b) Somewhat representative of the patients with COVID-19  c) Selected group of patients  d) No description of the derivation of the cohort | *****  ***** | 🞎  🞎  🞏  🞏 |
| 2. Selection of the non intervention cohort  a) Drawn from the same community as the intervention cohort  b) Drawn from a different source  c) No description of the derivation of the non intervention cohort | ***** | 🞎  🞏  🞏 |
| 3. Ascertainment of intervention  a) Secure record (e.g. health care record)  b) Structured interview  c) Written self report  d) Other / no description | *****  ***** | 🞏  🞏  🞏  🞏 |
| 1. Demonstration that outcome of interest was not present at start of study 2. yes 3. no | ***** | 🞏  🞏 |
| **Comparability** (tick one or all boxes, as appropriate) |  |  |
| 1. Comparability of cohorts on the basis of the design or analysis   1. Study controls for **Thyroxine Levels** 2. Study controls for **Severity of COVID-19** 3. Study controls for **COVID-19 or Non-COVID-19** 4. Study controls for **NTIS or Non-NTIS** | *****  *****  *****  ***** | 🞎  🞏  🞎  🞏 |
| **Outcome** (tick one box in each section) |  |  |
| 1. Assessment of outcome  a) Independent blind assessment  b) Record linkage  c) Self report  d) Other / no description | *****  ***** | 🞏  🞏  🞏  🞏 |
| 2. Was follow up long enough for outcomes to occur  a) Yes, if median duration of follow-up >= 3 month  b) No, if median duration of follow-up < 3 months | ***** | 🞏  🞏 |
| 3. Adequacy of follow up of cohorts  a) Complete follow up: all subjects accounted for  b) Subjects lost to follow up unlikely to introduce bias: number lost <= 20%,  or description of those lost suggesting no different from those followed  c) Follow up rate < 80% (select an adequate %) and no description of those lost  d) No statement | *****  ***** | 🞏  🞏  🞏  🞏 |

Note: A study can be awarded a maximum of one star for each numbered item within the Selection and Outcome categories. A maximum of two stars can be given for Comparability


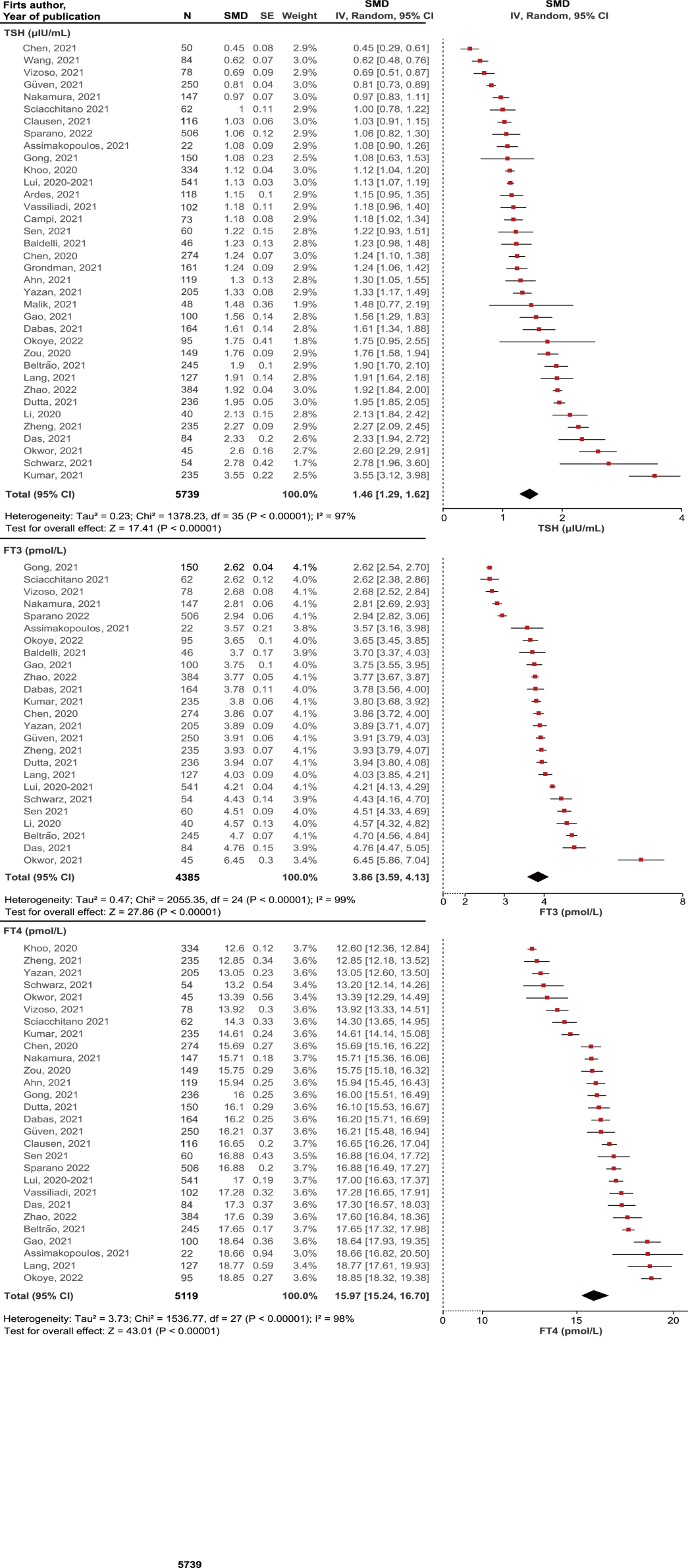


**eFigure 1. Forest plot for all studies comparing the Thyroxine levels at admission.**

**eFigure 2. Forest plot comparing the Thyroxine levels between Asian and European studies.**

**eFigure 3. Forest plot comparing the Thyroxine levels between non-COVID-19 pneumonia and healthy cohort in the COVID-19 pandemic.**


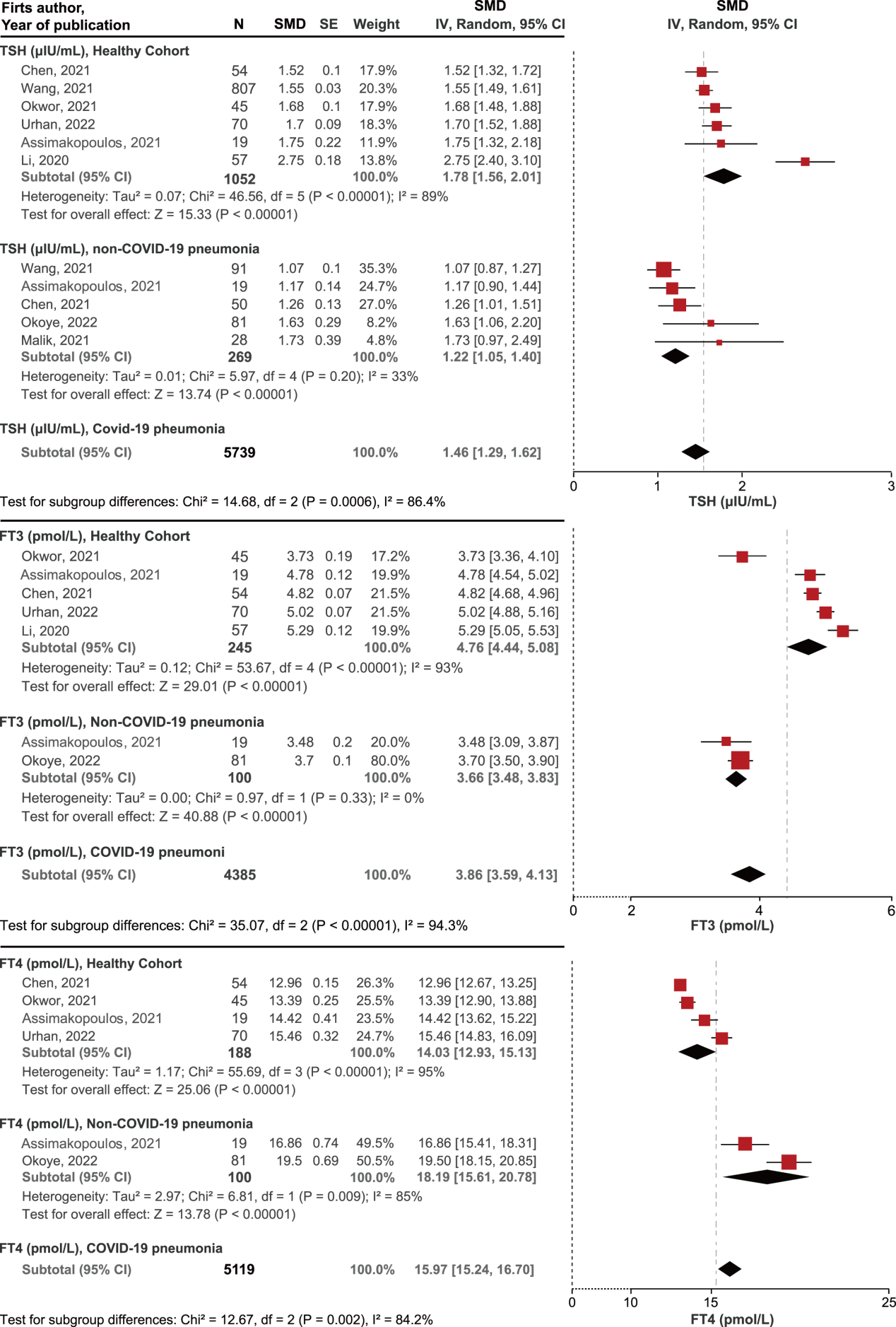


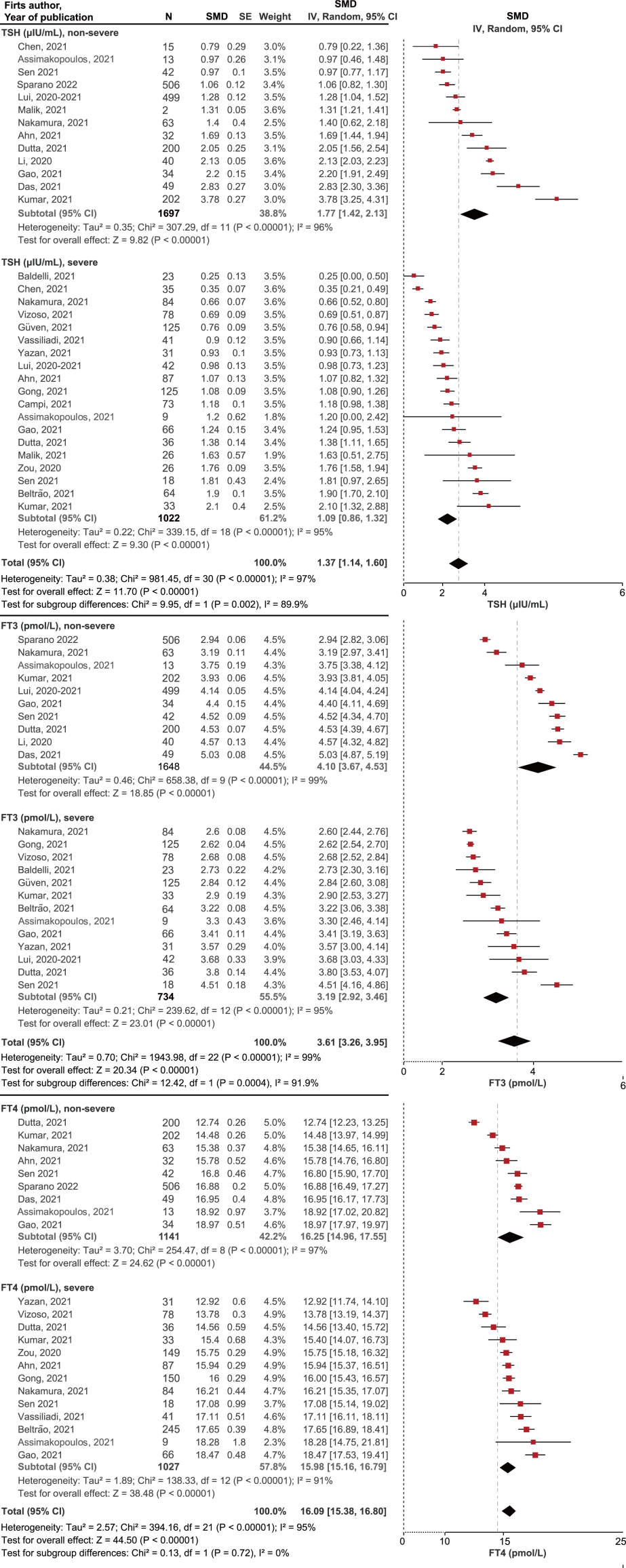


**eFigure 4. Forest plot for all studies comparing the Thyroxine levels in patients with different levels of COVID-19 severity.**

**eFigure 5. Forest plot comparing the different clinical classification of Thyroxine levels between the severe patients and nonsevere patients with COVID-19.**


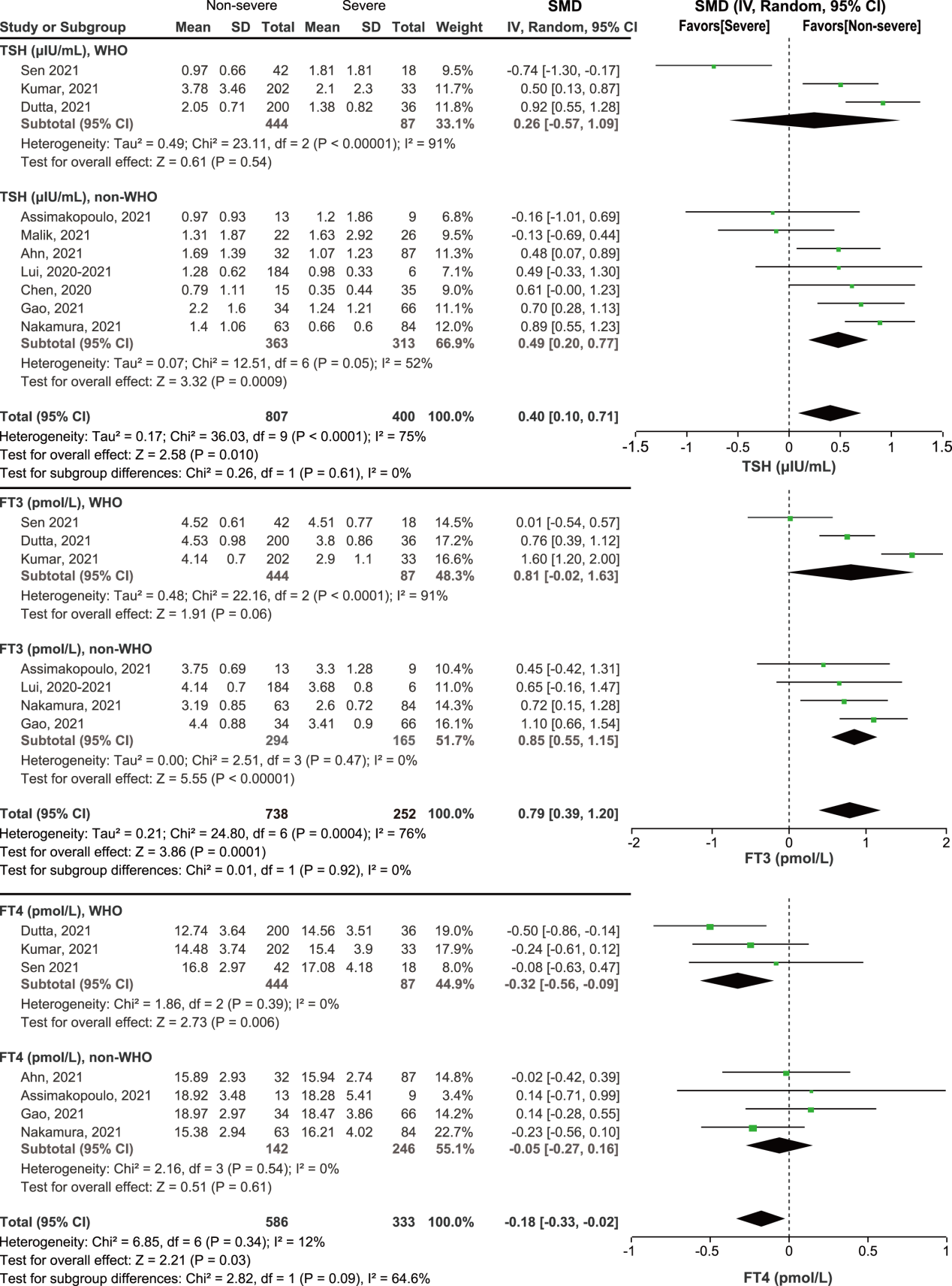


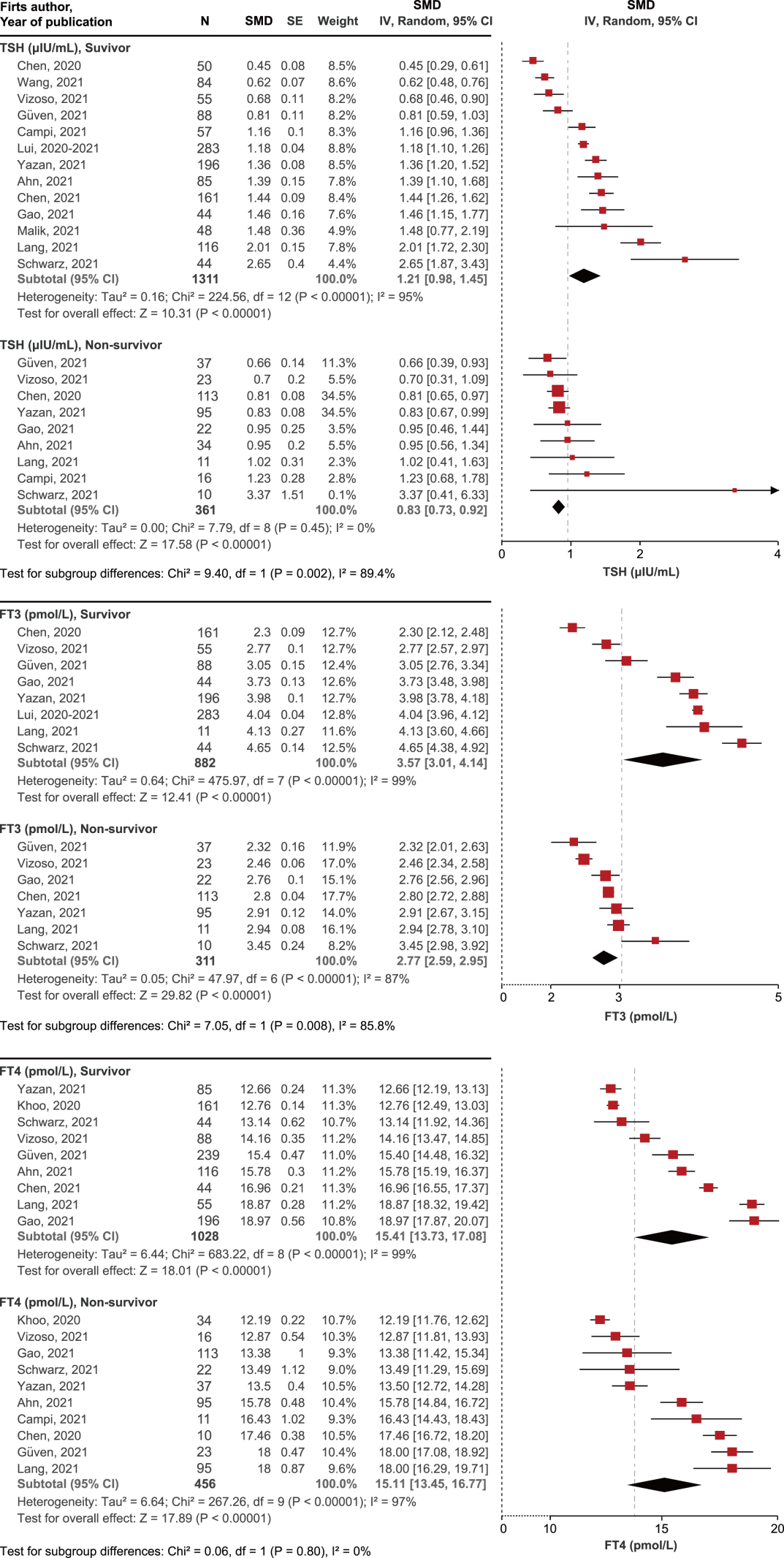


**eFigure 6. Forest plot for all studies comparing Thyroxine levels in different prognoses of patients with COVID-19.**

**eFigure 7.** Forest plot comparing the Thyroxine levels in different prognoses between ICU and All ward patients with COVID-19.


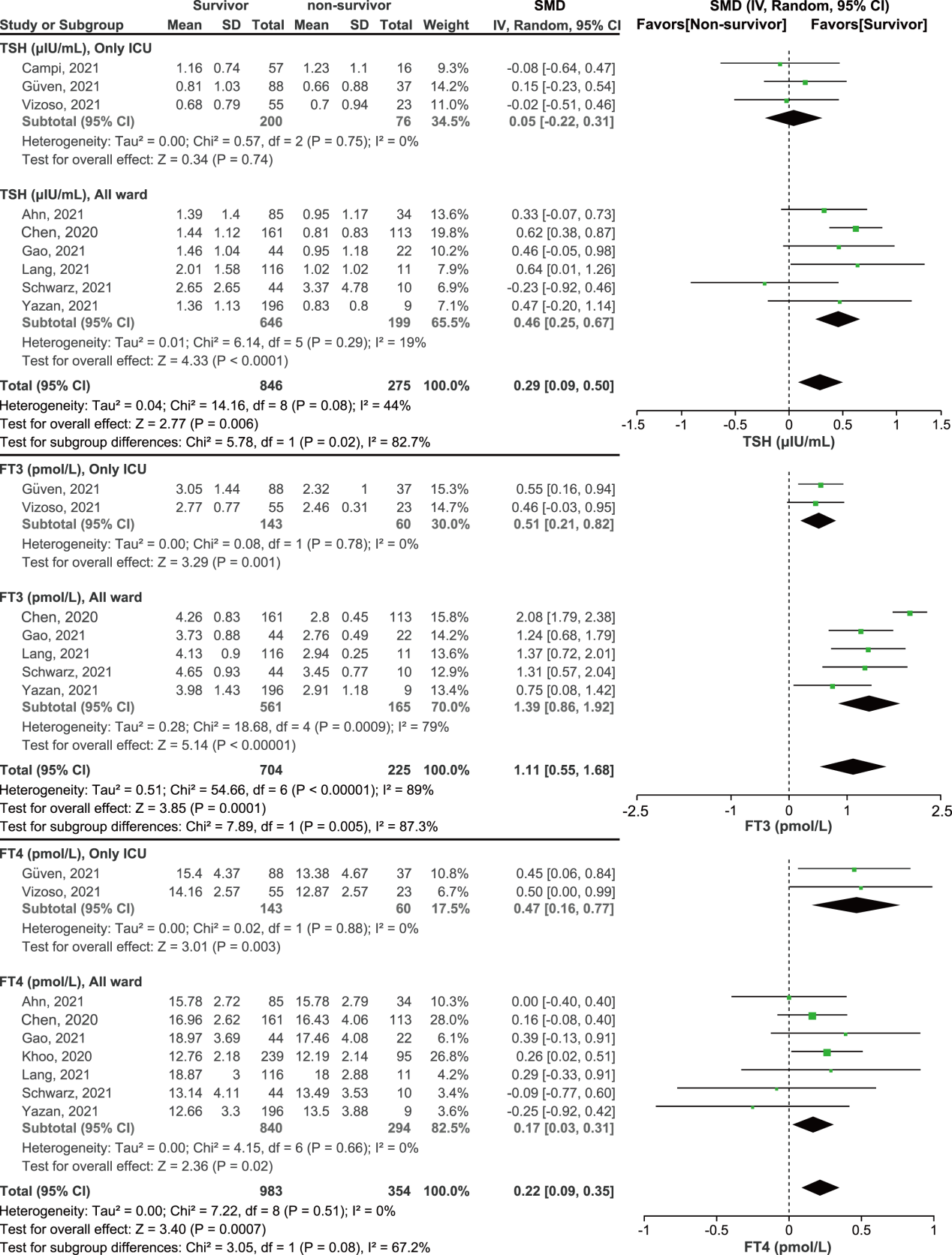


**eFigure 8. Forest plot comparing the Thyroxine levels during follow-up.**


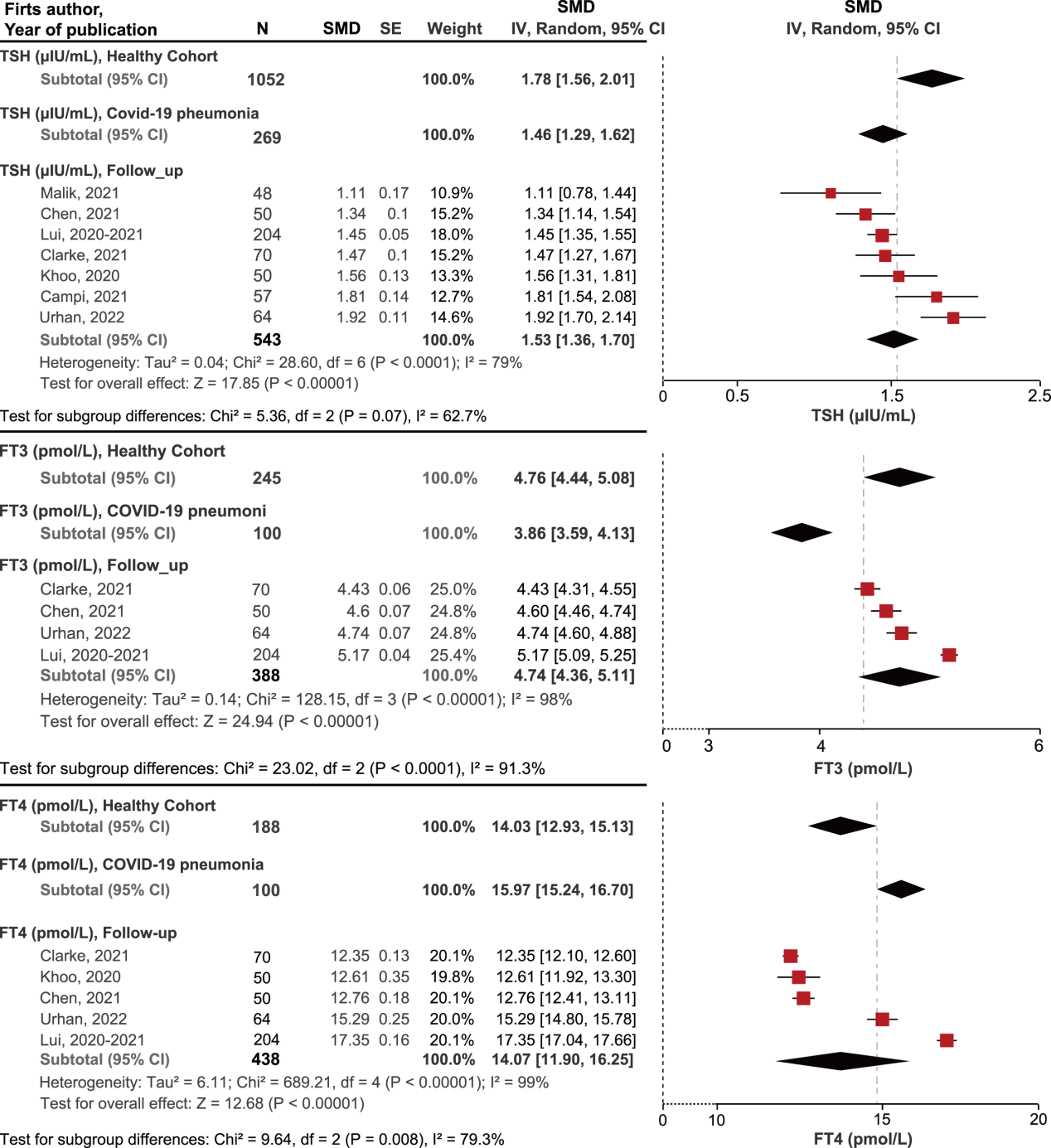

Supplement: Supplementary file 1 [file DataSheet_1.docx]
